# Impact of Supratentorial Cerebral Hemorrhage on the Complexity of Heart Rate Variability in Acute Stroke

**DOI:** 10.1038/s41598-018-29961-y

**Published:** 2018-07-31

**Authors:** Chih-Hao Chen, Sung-Chun Tang, Ding-Yuan Lee, Jiann-Shing Shieh, Dar-Ming Lai, An-Yu Wu, Jiann-Shing Jeng

**Affiliations:** 1 Stroke Center and Department of Neurology, National Taiwan University Hospital, Taipei, Taiwan; 2 Graduate Institute of Epidemiology and Preventive Medicine, National Taiwan University, Taipei, Taiwan; 3 NTU-NTUH-MediaTek Innovative Medical Electronics Research Center, Taipei, Taiwan; 4 Graduate Institute of Electronics Engineering, National Taiwan University, Taipei, Taiwan; 5 Department of Mechanical Engineering and Innovation Center for Big Data and Digital Convergence, Yuan Ze University, Taoyuan, Taiwan; 6 Division of Neurosurgery, Department of Surgery, National Taiwan University Hospital, Taipei, Taiwan

## Abstract

Acute stroke commonly affects cardiac autonomic responses resulting in reduced heart rate variability (HRV). Multiscale entropy (MSE) is a novel non-linear method to quantify the complexity of HRV. This study investigated the influence of intracerebral hemorrhage (ICH) locations and intraventricular hemorrhage (IVH) on the complexity of HRV. We recruited 93 supratentorial ICH patients (male 59%, mean age 61 years), and the locations of ICH included basal ganglia (n = 40), thalamus (n = 35), and lobar (n = 18) group. Continuous one-hour electrocardiography signals were obtained from patients after admission, and the complexity index was defined as the area under the MSE curve. The complexity index was lower in lobar ICH (21.6 ± 7.9) than basal ganglia (27.9 ± 6.4) and thalamus (28.5 ± 7.2) groups. The complexity index was inversely correlated with initial stroke severity (*r* = −0.26), size of hematoma (*r* = −0.35) and ICH score (*r* = −0.26), especially among patients with intraventricular hemorrhage (*r* = −0.60, −0.60, and −0.41 respectively). A higher complexity can predict a good functional outcome (adjusted odds ratio 1.09, 95% confidence intervals 1.00–1.19) at post-stroke 3 months. In summary, more severe stroke and larger hematoma volume resulted in lower complexity of HRV. Lobar hemorrhage and IVH had great impacts on the cardiac autonomic function.

## Introduction

The link between the brain and the heart is well recognized. For example, acute stroke commonly affects cardiac autonomic responses^1^. The proposed mechanisms for brain-heart interaction after stroke included activation of the hypothalamic–pituitary–adrenal axis, sympathetic and parasympathetic regulation, catecholamine surge, immune responses and inflammation^2^. Heart rate variability (HRV), an indicator of the heart’s ability to adjust instantaneously according to circulatory changes, can serve as an important parameter for the assessment of autonomic functions^3^. Studies have shown that a reduced HRV is commonly seen in patients after acute stroke, and can predict post-stroke mortality or poor outcome^4–7^. Since the central control of autonomic nervous system resides not only at the spinal cord or brainstem level, also among the widespread cortical and subcortical network including bilateral insular cortex, cingulate gyrus, amygdala and hypothalamus, whether the locations of acute stroke would have impact on the HRV is worth to be investigated^5,8^. Many studies explored the associations between infarct locations and the change of HRV or cardiac arrhythmia^4,5,9–12^, while less is known about the locations of intracerebral hemorrhage (ICH) and corresponding autonomic change^13–15^.

Conventional linear HRV analyses, such as frequency and time domain analyses, are usually applied on the studies of stroke related autonomic dysfunction^4,6,10^. Given that the modulation of HRV is thought to be originated from non-linear processes with non-stationary nature, properly use of the analyses based on chaos theory may be more reliable to describe the complex human biological signals^16–19^. Recently, multiscale entropy (MSE) has been developed as a non-linear method to quantify HRV^18,19^, and we have shown that early assessment of HRV by MSE can predict outcomes and potential stroke-in-evolution in patients with acute ischemic stroke^20,21^. Here, we hypothesized that different locations of the supratentorial ICH and the presence of intraventricular hemorrhage (IVH) could have contrasting impacts on the HRV. Both linear and non-linear methods would be applied to quantify the change of HRV.

## Methods

### Standard protocol approvals, registrations, and patient consents

The present study was approved by the Institutional Review Board of National Taiwan University Hospital to prospectively collect information on acute stroke patients, including stroke severity, risk factors, stroke mechanism, and outcome. All participants gave their written informed consent by themselves or from the next of kin of patients with impaired consciousness, and the methods were carried out in accordance with the approved guidelines.

### Study design, Setting, and Patients

Patients with acute non-traumatic supratentorial ICH who had been admitted within 24 hours to the stroke intensive care unit were recruited prospectively. Age- and sex-matched control participants free of acute cardiovascular or cerebrovascular events within 12 months were recruited from health check-up in cardiovascular outpatient clinic. Among patients with supratentorial ICH, their demographic data (age and gender), medical history, use of antiplatelet or anticoagulant agents, vascular risk factors, including hypertension, diabetes mellitus, dyslipidemia, atrial fibrillation, prior stroke, and history of smoking were recorded. Vital signs including blood pressure, heart rate, ECG, respiratory rate, and oxygen saturation were documented. The initial stroke severity was assessed by the National Institute of Health Stroke Scale (NIHSS) upon admission to the stroke ICU. Patients with modified Rankin scale (mRS) >2 prior to the index event, symptomatic heart failure, inability to obtain ECG signals within 48 hours of admission, or poor quality or artifacts of ECG signals were not included for analysis. We also excluded patients with atrial fibrillation since our previous study showed that the non-linear MSE analysis had poor predictive ability among this patient group^21^.

The diagnosis of ICH was confirmed by computed tomography (CT) of the head. The primary locations of ICH were classified into basal ganglia, thalamic, or lobar areas. The presence of intraventricular hemorrhage (IVH) was also documented. The volume of ICH was calculated based on the first CT scan using the (*a* × *b* × *c*)/2 method, where *a* and *b* represented the largest perpendicular diameters of the ICH zone, and *c* was the number of CT slices with hemorrhage multiplied by the slice thickness^22^. The severity of ICH was graded according to the well-known ICH score, ranged from 0 to 6 with higher score indicating higher 30-day mortality^23^. Since we only included the supratentorial ICH, the maximum of the ICH score of our patients was 5. A good function outcome was defined as mRS ≤2 at 3 months after stroke.

### ECG Data Acquisition and Analysis

Details of the method used for ECG data recording and analysis have been previously published^8^. Briefly, we collected ECG analogue data directly from the bedside monitor (Philips Intellivue MP70, Koninklijke Philips N.V., Amsterdam, Netherlands) for each patient. We collected the ECG data as soon as possible upon patient’s admission to the stroke care unit. To prevent the possible bias of data selection, only the first hour of the data was taken for analysis in all study subjects. One-hour ECG data were digitized with a sampling rate of 512 Hz and stored in a computer. For control participants, ECG signal was recorded via a Holter monitor (MyECG E3-80, Mircostar Company, Taipei, Taiwan). The stored ECG data then went through a pre-processing step to extract the R-R interval time series before analysis. To analysis the ECG data, we extracted the R-R interval of each patient in one hour first. Generally, there are more than 3600 intervals within 1 hour (about 4000~6000 intervals). We chose the first 3600 data for our analysis as 1 hour data. For 5 min processing, we segmented the 3600 points into 300 times 5 points without overlapping^24^. In addition, to ensure the stationarity of ECG signals utilized for further linear and nonlinear analyses, we performed the stationarity test by a previously described methods^25^. The results showed that the percentage of stationary segments is 70.35% (see Supplemental Fig. S1 for the detailed protocol).

The two main linear analyses of HRV included: (1) time-domain analyses including deviation of normal to normal R wave (SDNN) and root-mean-square of successive beat-to-beat differences (RMSSD); (2) frequency-domain HRV analysis including high frequency power (0.15–0.4 Hz), low-frequency power (0.04–0.15 Hz), and the ratio of low-frequency to high-frequency (LF-HF) power.

Non-linear MSE analysis was applied to the acquired 1-hour ECG data and it comprised two steps: (1) coarse-graining the signals into different time scales, (2) sample entropy calculation for each coarse-grained time series to quantify the degree of irregularity^12^ Then the entropy is calculated as a function of scale to provide a measure of information richness embedded in different time scales. We coarse-grained the original time series up to a scale factor of 20, which is a typical choice in most studies^18,21^. The parameters for sample entropy calculation were as below: embedding dimension was 2 (2 match over 3 match), tolerance was standard deviation times 0.2, time lag was 1 point, and the frame length was one hour which included at least 4000 R-R intervals. A detrending process was performed to attenuate the influence caused by non-stationary artifacts^17^. We calculated three different parameters derived from the MSE profile: the summations of quantitative values of scale 1–5 (Area_1–5_), scale 6–20 (Area_6–20_), and all scales (Area_1–20_, named the ‘complexity index’), which represented the short-term, long-term, and overall complexity, respectively^26^.

### Statistical Analysis

Among the three groups of ICH patients (basal ganglia, thalamic, and lobar), their demographic and clinical characteristics, medical history, volume of ICH, ICH scores, functional outcome, as well as the linear and non-linear parameters of HRV were compared using χ^2^ test, one-way ANOVA or Kruskal-Wallis test with relevant variables as indicated. Analysis of covariance (ANCOVA) was introduced to compare the significant differences of HRV between three groups after adjustment of age and sex. Similar analysis was performed to compare patients with or without IVH. The MSE curves of control and ICH patients were plotted and complexity index values were presented as the mean ± standard deviation.

To evaluate potential correlation between HRV parameters and certain important clinical variables, partial correlation between the value of each HRV parameter and initial NIHSS score, ICH volume and ICH score were tested by Spearman rank-order correlation after adjusted by age and sex. Whenever significant correlation between HRV parameters and above-mentioned clinical variables were found, further exploration by the location of ICH was performed.

We performed multivariable logistic regression models that were adjusted for age, sex, initial NIHSS score, and the presence of IVH to test for an association between good functional outcome and parameters of HRV (values of linear and non-linear parameters were entered as continuous variables). A *P* value of <0.05 was considered to indicate statistical significance. The SAS version 9.4 (SAS Institute Inc., Cary, NC, USA) was used for all analyses.

## Results

### Participants and Descriptive Data

There were 331 consecutive acute stroke patients admitted to the stroke intensive care unit received at least 1-hour ECG monitoring and HRV analyses between February 2012 and June 2014. We excluded 193 patients with ischemic stroke, 12 patients with diagnosis of non-stroke, and 5 patients with atrial fibrillation rhythm. Among the rest of 121 patients with ICH, 22 were further excluded because of their infratentorial origin of ICH, and 6 of multiple primary locations of ICH. A total of 93 patients with supratentorial ICH (male, 59.1%; age, 61.1 ± 15.3 years old) were finally included in the analysis, and were classified into basal ganglia (n = 40, 43.0%), thalamus (n = 35, 37.6%), and lobar area group (n = 18, 19.4%). Patients with lobar area ICH were significantly older and had larger ICH volume than those with basal ganglion and thalamic ICH, while patients with thalamic ICH more frequently had IVH (Table 1). The NIHSS score at admission and the ICH score, however, were similar between the three groups. Intraventricular extension of ICH was presented in 28 patients (30.1%), especially in the thalamus group (n = 22 of 35, 62.9%). Patients with IVH had higher initial NIHSS and ICH score, and were less likely to have a good functional outcome (Supplemental Table 1). In addition, 50 age- and sex-matched controls (male, 60%; age, 60.2 ± 10.8 years old) were recruited and received 1-hour ECG monitoring for HRV analyses.

**Table 1:** Characteristics of Patients with Supratentorial Intracerebral Hemorrhage by Lesion Locations.

	All	Basal ganglia	Thalamus	Lobar	*P* value^a^
Number	93	40	35	18	
Age, years	61.1 ± 15.3	56.8 ± 16.1	60.7 ± 14.0	71.2 ± 11.1	<0.01
Male	55 (59.1)	25 (62.5)	20 (57.1)	10 (55.6)	0.84
Hypertension	85 (91.4)	37 (92.5)	33 (94.3)	15 (83.3)	0.38
Diabetes mellitus	24 (25.8)	10 (25.0)	12 (34.3)	2 (11.1)	0.18
SBP at admission	176.7 ± 35.6	181.7 ± 35.8	175.3 ± 33.7	168.9 ± 38.7	0.43
NIHSS at admission	13.5 ± 8.1	13.5 ± 7.4	14.1 ± 9.1	12.5 ± 7.8	0.96
ICH volume	13.8 (5.2–33.1)	15.1 (6.0–30.5)	8.9 (3.3–19.3)	29.6 (15.9–51.4)	<0.01
ICH score	1.1 ± 1.3	0.9 ± 1.0	1.3 ± 1.2	1.2 ± 1.1	0.32
Presence of IVH	28 (30.1)	3 (7.5)	22 (62.9)	3 (16.7)	<0.01
Good functional outcome	40 (43.0)	22 (55.0)	12 (34.3)	6 (33.3)	0.13
Parameters of HRV					
Complexity index (Area_1–20_)	26.9 ± 7.4	27.9 ± 6.4	28.5 ± 7.2	21.6 ± 7.9	0.01
Scale_1–5_ (Area_1–5_)	5.9 ± 1.7	6.2 ± 1.6	6.3 ± 1.6	4.7 ± 1.8	0.01
Scale_6–20_ (Area_6–20_)	21.0 ± 6.0	21.8 ± 5.2	22.2 ± 5.9	16.9 ± 6.4	0.01
Sample entropy	1.14 ± 0.38	1.16 ± 0.33	1.22 ± 0.33	0.96 ± 0.50	0.05
SDNN	60.2 ± 37.2	62.0 ± 36.9	57.5 ± 40.5	61.5 ± 32.4	0.61
RMSSD	49.7 ± 51.3	47.1 ± 47.7	42.3 ± 55.2	69.7 ± 48.8	0.04
High frequency	417.8 ± 514.2	425.0 ± 434.0	426.8 ± 666.4	384.3 ± 330.3	0.67
Low frequency	480.2 ± 1078.7	500.9 ± 1099.0	435.6 ± 1262.0	520.7 ± 577.9	0.16
Low frequency to high frequency ratio	2.00 ± 1.19	2.02 ± 1.13	2.27 ± 1.22	1.44 ± 1.14	0.05

Data are expressed as mean ± standard deviation or n (%), except ICH volume is median (interquartile range).

HRV, heart rate variability; ICH, intracerebral hemorrhage; IVH, intraventricular hemorrhage; NIHSS, National Institutes of Health Stroke Scale; RMSSD, root-mean-square of successive beat-to-beat differences; SBP, systolic blood pressure; SDNN, standard deviation of normal to normal R wave.

^a^Compared between basal ganglia, thalamus and lobar group by one-way ANOVA or Kruskal-Wallis test.

### Main Results

**Figure 1:**
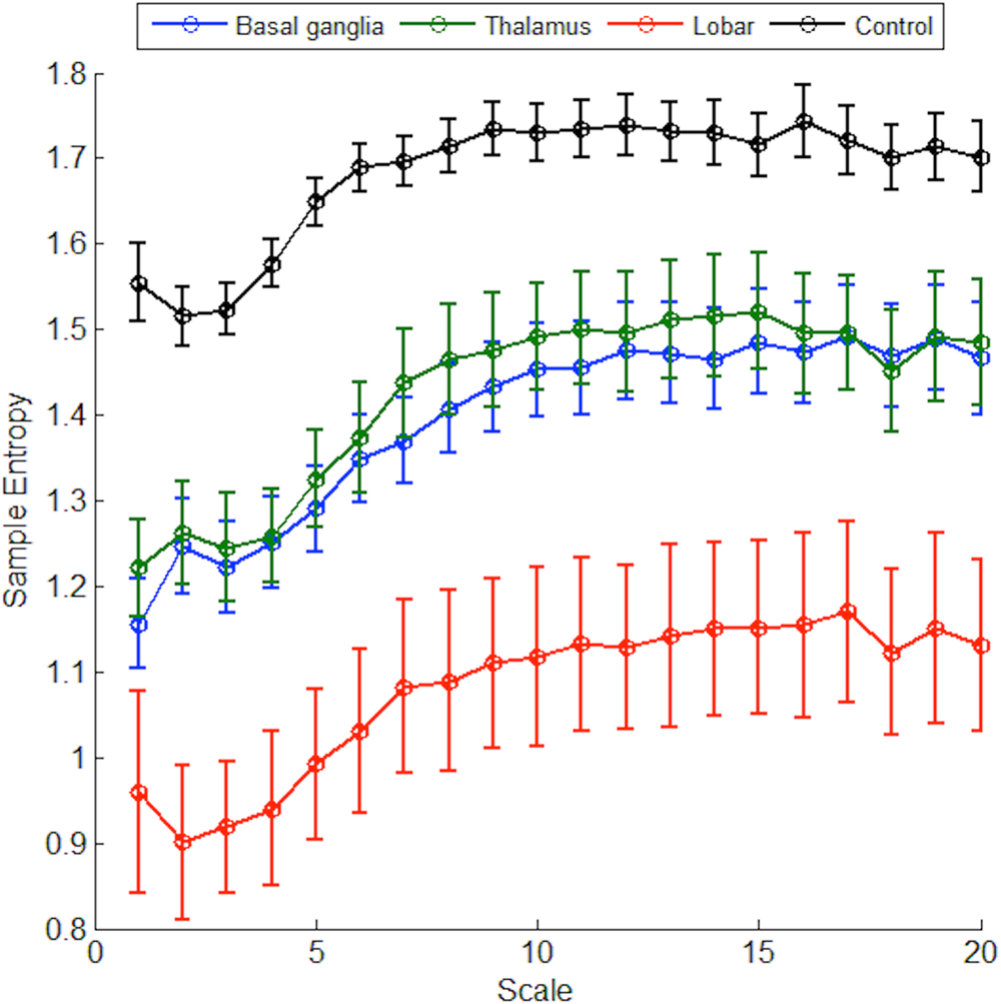
Plotted multiscale entropy (MSE) curves in patients with intracerebral hemorrhage (ICH) and controls The trends of MSE curves were apparently different from those obtained from supratentorial ICH patients (n = 93) and controls (n = 50), and lobar ICH had even lower complexity index than basal ganglia and thalamus ICH.

Figure 1 shows the plotted MSE curves in control and three groups of patients. The overall complexity index was significantly lower in patients with ICH than control group (26.9 ± 7.4 vs 33.6 ± 3.7, *P* < 0.001), and the curves were conspicuously lower in the lobar group. The linear parameters of HRV were mostly comparable between the three groups except RMSSD (*P* = 0.04) and LF-HF ratio (*P* = 0.05, Table 1). On the other hand, the values of complexity index derived from MSE were significantly lower in the lobar (21.6 ± 7.9) than the basal ganglia (27.9 ± 6.4) or thalamus groups (28.5 ± 7.2, overall *P* = 0.002). The results were consistent for the values of Area_1–5_ and Area_6–20_ (overall *P* = 0.002 and 0.004, respectively), while sample entropy (scale 1) showed borderline difference (*P* = 0.053) between three groups. After adjusted for age and sex, only the complexity index (Area_1–20_) remained statistical significance among three groups (*P* = 0.03), and other parameters did not. There was no significant difference between HRV parameters regarding to the presence of IVH or not (Supplemental Table S1).

After adjustment of age and sex, only the complexity index was negatively correlated with the NIHSS score at admission (*r* = −0.26, *P* = 0.01), ICH volume (*r* = −0.35, *P* = 0.0006) and ICH score (*r* = −0.26, *P* = 0.01; Table 2), while no significant correlation existed between the linear parameters of HRV and above clinical variables. In the lobar group, larger ICH volume and higher ICH score were significantly correlated with lower complexity index (*r* = −0.68, *P* = 0.004 and *r* = −0.59, *P* = 0.02, respectively). The significantly negative correlation between complexity index and NIHSS score at admission was only found in the thalamus group (*r* = −0.37, *P* = 0.03).

**Table 2:** Correlations Between Linear and Non-linear Parameters of Heart Rate Variability and Clinical Variables.

**Correlation coefficient** ***r*** **(** ***P*** **value)** ^a^	**NIHSS score**	**ICH volume**	**ICH score**
Complexity index	−**0.26** (**0.01)**	−**0.35** (**0.0006)**	−**0.26** (**0.01)**
Basal ganglia	−0.26 (0.11)	−0.20 (0.24)	−**0.37** (**0.02)**
Thalamus	−**0.37** (**0.03)**	−**0.34** (**0.05)**	−0.27 (0.12)
Lobar	−0.29 (0.28)	−**0.68** (**0.004)**	−**0.59** (**0.02)**
SDNN	−0.08 (0.43)	−0.09 (0.40)	−0.03 (0.78)
RMSSD	−0.13 (0.22)	−0.01 (0.95)	−0.03 (0.78)
High frequency	−0.13 (0.22)	−0.04 (0.71)	−0.03 (0.77)
Low frequency	−0.19 (0.07)	−0.08 (0.45)	−0.03 (0.73)
LF-HF ratio	−0.01 (0.93)	−0.03 (0.77)	−0.02 (0.86)

ICH, intracerebral hemorrhage; LF-HF, low-frequency to high-frequency; NIHSS, National Institutes of Health Stroke Scale; RMSSD, root-mean-square of successive beat-to-beat differences; SDNN, standard deviation of normal to normal R wave.

^a^Partial correlation was adjusted for age and sex.

Numbers in bold indicated significant findings.

When stratified by the involvement of IVH or not, the complexity index was strongly correlated with initial NIHSS score (*r* = −0.60, *P* = 0.001), ICH volume (*r* = −0.60, *P* = 0.001) and ICH score (*r* = −0.41, *P* = 0.04) among patients with IVH, while the negative associations were less prominent in those without (*r* = −0.09, *P* = 0.48; *r* = −0.26, *P* = 0.04; *r* = −0.26, *P* = 0.04, respectively; Supplemental Table S2).

In the multivariable logistic regression model after adjustment for age, sex, NIHSS score at admission and the presence of IVH, higher complexity index was a predictor of a good functional outcome (OR = 1.09, 95% confidence intervals = 1.00–1.19, *P* = 0.039; Table 3). We did not further explore this association in different groups because of their relatively small sample sizes.

**Table 3:** Adjusted Linear and Non-linear Parameters of Heart Rate Variability in predicting functional status 3 months after stroke.

	Good outcome	Poor outcome	Adjusted OR (95% CI)^a^
Complexity index	29.8 ± 5.6	24.8 ± 8.0	1.09 (1.00–1.19)
SDNN	63.9 ± 36.7	57.4 ± 37.6	1.01 (0.99–1.03)
RMSSD	46.4 ± 48.3	52.1 ± 53.8	1.00 (0.99–1.02)
High frequency	436.8 ± 1174.5	492.5 ± 1011.9	1.00 (1.00–1.00)
Low frequency	490.7 ± 644.2	362.8 ± 386.5	1.00 (1.00–1.00)
LF-HF ratio	2.09 ± 1.15	1.94 ± 1.23	1.06 (0.66–1.69)

CI, confidence intervals; LF-HF, low-frequency to high-frequency; OR, odds ratio; RMSSD, root-mean-square of successive beat-to-beat differences; SDNN, standard deviation of normal to normal R wave.

^a^Logistic regression was adjusted for age, sex, NIHSS score at admission and presence of intraventricular hemorrhage.

Numbers in bold indicated significant findings.

## Discussion

This study explored potential effect of the locations and severity of supratentorial ICH on various parameters of HRV. Patients with lobar ICH had the lowest complexity of HRV derived from non-linear method of MSE. The complexity index was negatively correlated with ICH volume and clinical severity (the NIHSS score at admission and ICH score), especially in the lobar ICH or those with intraventricular extension of hemorrhage. Our study clearly demonstrated that the non-linear method of MSE is superior to conventional linear analysis of HRV on reflecting the clinical severity and functional outcome of ICH.

Among linear parameters of HRV, only the time series of RMSSD and frequency domain of LF-HF ratio showed significant change in lobar group. Traditionally, RMSSD indicated vagal modulation of short-term, rapid changes of heart rate variability^27^, whilst LF-HF ratio implied the balance between sympathetic (LF) and parasympathetic (HF) balance on the cardiac autonomic function^4^. In the present study, lobar group had a higher crude RMSSD value and lower LF-HF ratio, possibly suggesting a stronger vagal and less sympathetic drive in these patients. However, the significant findings diminished after introducing covariates (age and sex), and these parameters were not associated with clinical severity and outcome either. The authors therefore focused mainly on nonlinear parameters of HRV in this study.

The reasons why patients with lobar ICH had lowest complexity of HRV might be multifactorial. Larger hematoma size and older age per se could result in lower complexity^19^. In addition, it could be caused by damage to the central control of autonomic nervous system, which was thought to be locating in insular cortex, cingulate gyrus, amygdala and hypothalamus^5,8^. Of these locations, insular cortex was the most comprehensively studied area, and there were controversies regarding whether left or right side was ‘dominant’ for sympathetic or parasympathetic responses^10,12,28–31^. These conflict results, however, should be interpreted carefully since the cardiovascular autonomic center might not be exactly located within the insula but also involved extra-insular areas and their interconnecting fibers^11^, and the findings might also be somehow deviating based on lesioning or stimulating studies^32,33^. Hence, we simplified this issue and sorted lobar-origin ICH in a group. Given the small sample size within lobar group, we did not further differentiate those involving insular or lateralization. At least the authors observed that more severe destruction of lobar area by larger hematoma size resulted in more depressed of the HRV, which also reflected on the worse clinical outcome.

The common non-lobar, supratentorial locations of ICH involved basal ganglia and thalamus^34^. We found no significant differences existed between the parameters of HRV, stroke severity and functional outcome among these two groups, which echoed previous studies^34^. One important difference between these two groups, however, was that more intraventricular extension of bleeding occurred in the thalamus group. In our study, although unfavorable outcome was more prevalent in patients with IVH, the complexity index or other parameters of HRV were not different between those with and without IVH. The lack of further discrimination of locations of IVH possibly contributed to this result. Nonetheless, the negative correlations between the complexity index and clinical indicators were more salient in patients with IVH. One study had demonstrated that hematoma extension to the third and fourth ventricles may cause an impaired baroreflex sensitivity^15^. Since there is a rich network structure controlling autonomic function surrounding the third and fourth ventricles, including hypothalamic paraventricular nucleus, circumventricular organs in the anterior wall of the third ventricle, periaqueductal gray matter, brain stem and medullar nuclei^8^, intraventricular extension of hematoma might cause pressure disturbance on these periventricular controlling centers and result in autonomic dysfunction.

The study found that higher complexity index can predict a favorable outcome among patients with ICH, which was consistent with our previous work^21^. In addition, more severe stroke, assessed by NIHSS and ICH score, also correlated with lower complexity index. One recent study demonstrated that SDNN, deceleration and acceleration capacity of heart rate were negatively correlated with the NIHSS score in patients with acute hemispheric ischemic stroke, though the values of correlation coefficient were rather low^35^. In our study on the ICH population counterpart, only the non-linear parameters negatively correlated with the severity of stroke, while linear parameters were not. Our results were more solid given that the *r* values were age- and sex-adjusted, which suggested that stroke severity could unambiguously influence the cardiac autonomic function. We also showed that the larger volume of the ICH, the more depressed of the complexity. Larger ICH usually corresponded to more tissue damage and higher clinical severity, hence more disturbance on the cardiac autonomic function could be expected. These correlations were especially evident in the lobar group and in those with IVH, implying the critical role of autonomic control around the cortical as well as periventricular areas.

This study had several limitations. First, our sample size was relatively small and derived from a single hospital. We only included supratentorial ICH in order to conveniently calculate the volume of ICH. Given this relatively few number of patients, we did not further explore the impact of lateralization and insular involvement on the HRV. We also could not rule out the possibility that the change of HRV was simply the sole effect of ICH in the absence of any involvement of autonomic areas in the brain. In addition, we chose control participants free of cardiovascular disease from outpatient clinic instead of patients admitted to ICU without ICH, which might amplify our results on the differences of HRV. However, our study aim focused on whether stroke location might affect the HRV, and patients with supratentorial ICH formed a unique population since the exact locations and size of brain insults can be easily calculated from simple neuroimaging study.

Secondly, despite that MSE has become a prevailing technique to quantify the complexity of physiological signals, it has several inherent methodological limitations, including artificially reduction of entropy due to the coarse graining procedure, inaccurate entropy estimation at large time scale, elimination of fast temporal scales and its introduction of spurious MSE oscillations, or even undefined entropy value. In order to overcome these drawbacks, several refined methods had been proposed^36,37^ and might be applied in our further clinical studies.

Thirdly, we excluded patients with AF rhythm because our previous study had shown that the value of the complexity index was less reliable in subjects with AF^21^. Interestingly, we recently confirmed the usefulness of photoplethysmogram signals from routine pulse oximetry in identifying AF rhythms^38^. Another study showed that systolic arterial blood pressure variability can also be powerful in predicting outcome in critical care^39^. Incorporating multi-parameter monitoring in the future can broaden the usefulness of autonomic function in critical settings. Last, we did not have the detailed image data with regard to cerebral microbleeds since the different locations of microbleeds might reflect distinct underlying pathophysiology, such that lobar origin are generally attributed to cerebral amyloid angiopathy while deep origin to hypertensive vasculopathy^40^.

## Conclusions

Our study demonstrated that non-linear complexity of HRV was related to stroke severity, size of hemorrhage and function outcome in patients with supratentorial ICH, especially with coexistence of IVH. Patients with lobar ICH had lowest complexity of HRV. These findings reinforced the concepts that acute stroke, especially those involving lobar areas, had great impact on the brain-heart axis and cardiac autonomic modulation, and a lower complexity index of MSE may played a novel predictor for outcome.

## Electronic supplementary material

Supplementary Information
