# Supplementary material for: Impact of Supratentorial Cerebral Hemorrhage on the Complexity of Heart Rate Variability in Acute Stroke
Source: Sci Rep. 2018 Jul 31;8:11473. doi: 10.1038/s41598-018-29961-y (PMC6068137; doi:10.1038/s41598-018-29961-y)
Supplement: Supplementary file 1 — Supplementary Information [file 41598_2018_29961_MOESM1_ESM.pdf]

Impact of Supratentorial Cerebral Hemorrhage on the Complexity of Heart Rate Variability in Acute Stroke

Chih-Hao Chen[1,2], Sung-Chun Tang[1,3*], Ding-Yuan Lee[4], Jiann-Shing Shieh[5], Dar-Ming Lai[3,6], An-Yu Wu[3,4], Jiann-Shing Jeng[1]

[1] Stroke Center and Department of Neurology, National Taiwan University Hospital, Taipei, Taiwan

[2] Graduate Institute of Epidemiology and Preventive Medicine, National Taiwan University, Taipei, Taiwan

[3] NTU-NTUH-MediaTek Innovative Medical Electronics Research Center, Taipei, Taiwan

[4] Graduate Institute of Electronics Engineering, National Taiwan University, Taipei, Taiwan

[5] Department of Mechanical Engineering and Innovation Center for Big Data and Digital Convergence, Yuan Ze University, Taoyuan, Taiwan

[6] Division of Neurosurgery, Department of Surgery, National Taiwan University Hospital, Taipei, Taiwan

Supplemental Figure S1. Protocol of stationarity test

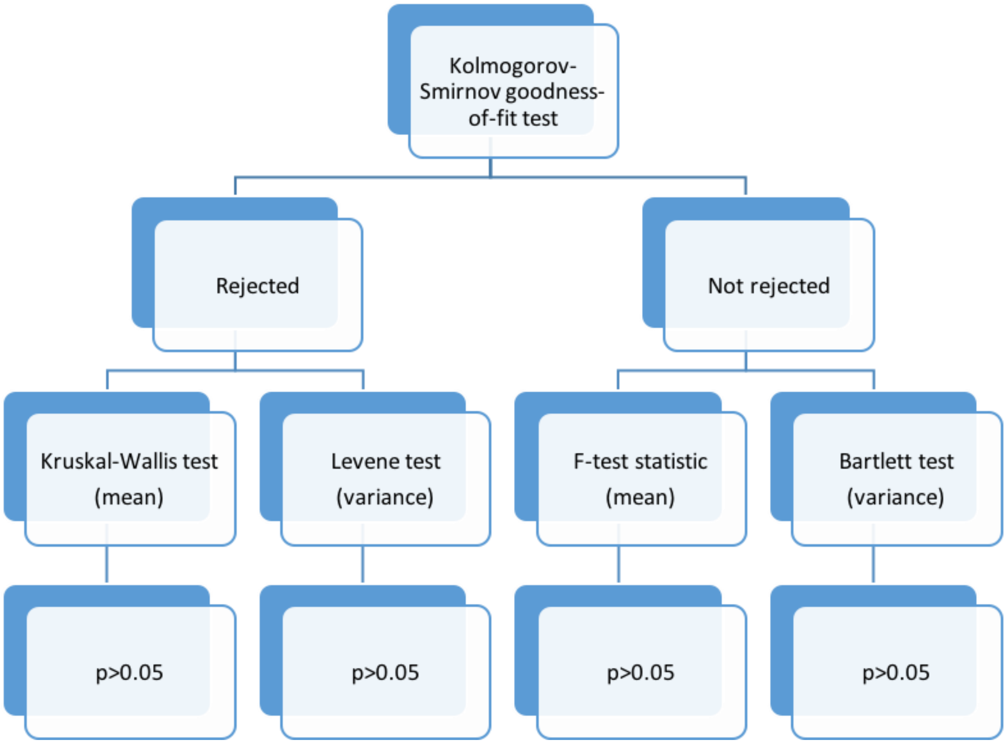

We performed the stationarity test according to Porta's method (see Porta et al., Comput. Cardiol. 31, 645-648, 2004). If *P* value of both mean and variance were >0.05, then the segment was considered stationary. After calculation, the percentage of stationary segments is 70.35%

Supplemental Table S1. Linear and Non-linear Parameters of Heart Rate Variability According to the Presence of Intraventricular Hemorrhage

	**IVH (+)**	**IVH (-)**	***P* value**
Number	28	65	
Age, years	64.0 ± 14.6	59.8 ± 15.5	0.23
Male	15 (53.6)	40 (61.5)	0.47
Hypertension	26 (92.9)	59 (92.2)	0.91
Diabetes mellitus	7 (25.0)	17 (26.6)	0.88
SBP at admission	175.4 ± 37.3	177.3 ± 35.0	0.81
NIHSS at admission	17.6 ± 6.9	11.8 ± 7.9	0.001
ICH volume	13.4 (8.4 – 43.5)	13.8 (3.8 – 27.3)	0.67
ICH score	2.1 ± 1.0	0.7 ± 0.9	<0.0001
Good functional outcome	6 (21.4)	34 (52.3)	0.01
Complexity index ($Area_{1-20}$)	26.0 ± 8.3	27.3 ± 7.1	0.49
$Scale_{1-5}$ ($Area_{1-5}$)	5.8 ± 1.8	6.0 ± 1.7	0.61
$Scale_{6-20}$ ($Area_{6-20}$)	20.2 ± 6.7	21.3 ± 5.7	0.54
SDNN	65.3 ± 46.8	58.0 ± 32.4	0.75
RMSSD	57.1 ± 70.4	46.5 ± 40.8	0.99
High frequency	690.1 ± 1606.4	398.7 ± 745.9	0.69
Low frequency	537.8 ± 817.4	366.1 ± 298.7	0.72

LF-HF ratio	1.97 ± 1.25	2.02 ± 1.18	0.77

Data are expressed as mean ± standard deviation or n (%), except ICH volume is median (interquartile range).

ICH, intracerebral hemorrhage; IVH, intraventricular hemorrhage; LF-HF, low-frequency to high-frequency; RMSSD, root-mean-square of successive beat-to-beat differences; SBP, systolic blood pressure; SDNN, standard deviation of normal to normal R wave.

Supplemental Table S2. Correlations Between Linear and Non-linear Parameters of Heart Rate Variability and Clinical Variables, according to the presence of intraventricular hemorrhage

With IVH (n=28)

**Correlation coefficient *r* (*P* value)** [a]	**NIHSS score**	**ICH volume**	**ICH score**
Complexity index	**−0.60 (0.001)**	**−0.60 (0.001)**	**−0.41 (0.04)**
SDNN	−0.19 (0.35)	−0.07 (0.74)	−0.15 (0.46)
RMSSD	−0.19 (0.35)	−0.21 (0.31)	−0.08 (0.68)
High frequency	−0.33 (0.11)	−0.18 (0.37)	−0.07 (0.73)
Low frequency	**−0.39 (0.05)**	−0.07 (0.73)	−0.01 (0.96)
LF-HF ratio	0.18 (0.38)	−0.20 (0.32)	−0.03 (0.89)

Without IVH (n=65)

**Correlation coefficient *r* (*P* value)** [a]	**NIHSS score**	**ICH volume**	**ICH score**
Complexity index	−0.09 (0.48)	**−0.26 (0.04)**	**−0.26 (0.04)**
SDNN	−0.09 (0.48)	−0.17 (0.19)	−0.07 (0.58)
RMSSD	−0.17 (0.19)	−0.10 (0.45)	−0.02 (0.85)
High frequency	−0.13 (0.29)	−0.14 (0.29)	−0.06 (0.62)
Low frequency	−0.18 (0.15)	−0.17 (0.18)	−0.08 (0.54)
LF-HF ratio	−0.01 (0.94)	−0.03 (0.83)	−0.03 (0.83)

ICH, intracerebral hemorrhage; IVH, intraventricular hemorrhage; LF-HF, low-frequency to high-frequency; NIHSS, National Institutes of Health Stroke Scale; RMSSD, root-mean-square of successive beat-to-beat differences; SDNN, standard deviation of normal to normal R wave.

[a] Partial correlation was adjusted for age and sex.

Numbers in bold indicated significant findings.
